# The Importance of Establishing a Higher Standard of Preliminary Education for Students of Medicine*President’s Address: Medical Society of North Carolina. Dr. Register is editor of The Charlotte Medical Journal.

**Published:** 1906-09

**Authors:** Edward C. Register

**Affiliations:** Charlotte, N. C.


### Abstracts and Selections

### The Importance of Establishing a Higher Standard of Preliminary Education for Students of Medicine.*

BY EDWARD C. REGISTER, M. D., CHARLOTTE, N. C.

The eminent men, who, through your confidence and through your courtesy and good will, have been permitted to occupy this position, have referred to the honor of this office, to the responsibilities attached to it, and, most feelingly, to those who have preceded them.

When I recall to mind these beautiful references, when I think of the brilliant men who have been president of this society, men who have preceded me, I feel more than ever my anxiety and my timidity, the need of your assistance and your advice.

There has never been a time in the history of this organization or in the history of medicine when medical progress was more active than it is now, when facts and theories were appearing so fast. One is startled at what he has missed if he fail to read medical journals and attend medical society meetings for only a few months.

Since the relation of bacteria to disease has been discovered much of pathology has been revolutionized and many changes in the treatment of medical and surgical cases have followed. Our abiilty to observe has been greatly aided by the numerous mechanical and physical means now at our disposal. With these means a correct diagnosis of many diseases once obscure is now easy, their pathology understood and the treatment simplified and effective. Chemical discoveries are now throwing new light on many physiological processes and appropriate therapeutic agents are fast multiplying. Specialists in the several branches of medicine and surgery are being developed and fostered; they have

*President's Address: Medical Society of North Carolina. Dr. Register is editor of The Charlotte Medical Journal.

their organizations and departments of investigation; they are achieving results that arouse our admiration and enthusiasm. Without their skill and without their accomplishments many cases would be neglected and many lives sacrificed that are now relieved and made strong.

Within the past two decades, through the aid and influence of a few of our great men, many special facilities for original research have been established. We can think of no beneficence from which greater good can emanate than from laboratories and establishments of this kind where scientific principles are coined and where we have a basis to achieve a more perfect technical development. For several years the activities of the medical profession have not been confined to medical principles alone. We find that through the influence of medical organizations like this and through their energy and wise direction many laws for the protection of the public and for the prevention of many kinds of disease and for the care and cure of the afflicted have been enacted. Scattered through nearly every paragraph of these enactments we see evidences that our legislative committee has done a great deal to elevate the standard of medical education, to make effective our sanitary and quarantine regulations and prevent the adulteration of food and drink and to care for the insane and feeble-minded.

It has occurred to many of us, no doubt, that our lay friends, the general public, do not, every time, understand our efforts, the high motives of physicians, or appreciate their sacrifices. Prominent officials sometimes depreciate our work and do not interpret correctly our motives. It would be much better for the people of the State if some of our judges could be made to believe that our methods tend to better principles; to higher ideals, and to a more perfect professional development.

My address, therefore, will, in a way, deal with the relation of this society to the people of the State.

Socially, and in a way professionally, each of us establishes our own social and professional position:—this we will not discuss. It is our relation to the State collectively as a body, that we are especially interested. Our united efforts, which are the effects of this body, have accomplished a great deal.

In 1858 our General Assembly enacted a law creating the Board of Medical Examiners of North Carolina and entitling only licensed physicians to testify in our courts, as experts. This was the beginning of medical legislation in this State.

In 1885 the law was amended in such a way that it was a misdemeanor to practice medicine in the State without first obtaining a license from our Medical Examining Board, and in 1897 all applicants for examination before this Board had to possess a diploma from a reputable school.

The establishment of a Board of Health with sanitary and quarantine regulations is still further evidence of the important relations we have created, and that now exist, between this society and the people of this commonwealth.

While we have through this body brought about these essential relations we are still far from an ideal relationship. When a member of the Medical Examining Board I noticed, and my associates on the Board also observed, that a very large per cent of the applicants for license showed many evidences that they were not primarily prepared to begin the study of medicine. Many of these young men seemed to be competent professionally and well trained technically, but their literary, pre-medical qualifications were very defective. It was perfectly plain that we were admitting, to what we always considered a learned profession, men who were not learned, men who were not educated, and men who will, with a notable exception occurring now and then, go through their professional lives laboring under many disadvantages. They are unfit to represent the profession before the people. They can not, and never will, conceal their literary defects. They have not the kind of knowledge to recognize their mistakes, in themselves. Their efforts will always be depreciated and they will never have the applause and the support of the thoughtful and cultivated.

The medical profession of North Carolina, if it wants to keep in line with other States and other countries, ought to undertake to correct these defects in our system; legislative changes should be advised. We need, and it is practical for us to have, medical laws created that provide that it shall be essential for a young man beginning the study of medicine, or before he can obtain his license, to have an essential, non-professional knowledge, certainly to the point where he can speak and write the English language correctly.

The Council of Medical Education, created by the American Medical Association, has made many valuable suggestions concerning the reforms necessary in medical training, particularly that which pertains to the elementary qualifications of medical students. This council has had several conferences with delegates from the different States and Territorial licensing boards, representatives from the associations of medical colleges, from the government medical services, and eminent men who represent colleges of the liberal arts. These conferences have been well attended and they have been regarded as a distinct success. At the Chicago conference, held some time ago, reports of several committees on preliminary education, on accessory technical students, were read to furnish a basis for discussion. As a result of these discussions the American Medical Association, at its approaching meeting in Boston, will very likely take steps to encourage the creation of laws in each State that will provide that all students beginning the study of medicine shall have at least a high school education, or such training as will admit them to our recognized universities, these qualifications to be passed on by specially designated State authorities, such as the superintendent of public instruction, or his representatives, and not by the faculty of a medical school. This is what we want here in North Carolina; it is what we we need, and we ought to make the change without the suggestion or aid of outside influences.

This council wisely concludes, after carefully considering the matter, that a discussion of reciprocity is not at this time advisable. There is not so much difference between the technical qualifications required by the different boards of the different States as there is in the minimum standard of the entrance examinations in the different schools.

The chief functions of all important medical associations should be the elevation of medical standards, the promotion of a higher professional education, and it should be the avowed purpose of this society to secure within a reasonable time as high a minimum standard of medical training as that of any State or any country in the world. Our position as a civilizing power and our position in commerce and our relation to the arts and to the sciences demand this of American medicine. An elevation from the present condition to a higher minimum standard that we advise, ought to be brought about slowly in justice to all concerned.

Many of us have been taught to believe that North Carolina is in the lead of all other States in perfecting legislative enactments bearing upon the practice of medicine, and that the United States is leading the world in medical progress. This may be so in many departments, but it is not so in others. These changes that I have roughly outlined are already practical laws in several States. These ideas were not original with these people. They got them from other countries. There is not a nation in the world that pretends to be civilized that does not have a system to determine when a young man is capable of beginning his professional training. Even Japan, many years before she was considered civilized by our international legations, had a system that clearly set forth the conditions, with which all who contemplated the study of medicine had to comply. And Russia, a country that has, according to our advices, a very unstable and corrupt government, is well regulated along these lines. Why is it, then, that our people who are so progressive and so energetic a class of people, who easily lead the world in commerce, in great financial enterprises, should be so indifferent to measures that are valued so highly by the same class in nearly every other country?

In the United States there has been a tendency for many years to increase the time devoted to medical training proper. This inclination has not been confined to medicine alone; the same influence has been noticed in all the professional schools. Possibly it is equally as conspicuous in the various lines of engineering and technology, or the departments that equip men for work in trade or commerce. In all these spheres of human activity the influence of modern scientific studies has been felt. If the physician wishes to obtain a perfect technical development, if he desires to keep in touch with the progressive ideas that are so much in evidence in all of the other professions, if he intends to be familiar with, and master the methods and principles of medicine and surgery, and if he expects to be able to think and to grasp complicated ideas, to be a leading citizen and a scientific physician, his training must be thorough, and it can not be thorough unless he has a general and liberal knowledge prior to the study of medicine. Of all the students and members of the so-called learned professions, it is necessary for the physician to be trained in more kinds of scientific study than any other man. To begin with: he must know the English language; he ought to have a knowledge of the classics, and he must know something of physics and a great deal about chemistry, and, technically, his studies must include many different sciences. Every advance in any of the sciences of medicine or surgery increases the importance of a more perfect preliminary qualification. There is a belief, that may be correct, that this training, which seems to be so essential to the successful practice of medicine and surgery, has, in many parts of the country and among some of the students of this State, reached a satisfactory point. This conclusion is plausible when we think of the time that must be devoted to the study of all of these essential sciences and that when a student incorporates a college course of the old type with his technical education the greatest efforts of his life have been exerted before he begins the practice of medicine. To insist on a uniform high standard that includes a preliminary college course of the old type is not practical and will do harm. It is evident, therefore, that we have to deal with conflicting ideas as to the relative value of a high standard of general education or a high standard of the technical sciences or both. This conflict of ideas involves many different issues and deals with methods of much perplexity. When we compel our young men to give over four years of their time to professional training we are apt, in many cases, to observe that their general knowledge has been neglected. Especially is this so if the former is compulsory and the latter optional. It may be well enough to have a very high professional standard, as it is here in North Carolina, but if we advocate a still higher standard and neglect the college or high school training, that is so essential to the professional man, we are apt to make a mistake.

I admit that modern medical colleges incorporate so many of the sciences that are at the foundation of the study of the principles of medicine and surgery, that it has in a way, reversed the old order of things, and it is not so essential for the student of medicine to spend as much time in the high school or college as it once was.

If our professional schools continue to increase their curriculum until every science and enough of the arts are taught that are essential to thoroughly equip the young medical man, then the question, as many consider it, will be solved, but now we seem to be a long way from this ideal. There is a belief that the young man is better equipped if he acquires as much of his professional knowledge as is available outside of the medical school proper. All of our universities and many of our colleges are well prepared to teach the sciences that ought to be the basis for the successful study of medicine. Here they are in an ethical atmosphere that will broaden their views. They are amid a set of associates that bring them in contact with non-professional life, on as many sides as possible. On the other hand, in the medical school they are in an atmosphere that reduces these outside influences to the minimum and encourages them to narrow their efforts to strictly professional thought.

When this plan prevails, when the student obtains his knowledge from a strictly professional institution, he has not the accomplishments, the breadth and tact to deal as successfully with the social and semi-medical problems that come up every day in the medical man's life, as the student of medicine, who has had different training and different environments. Of course, it is generally known that knowledge is more specific when obtained in a technical school with few and simple surroundings, but specific information, when it takes the place of knowledge of the principles of things, is not of as much practical value.

We have fallen into the error of believing that men of their own accord, without being forced to do so, will acquire a general knowledge before taking up even the initial sciences of medicine. We have hundreds of examples to show that they will not. The per cent of young medical men now entering the profession who can not enter the eighth grade in our public schools is very large. I believe that every member of the examining board of this State, certainly those who were associated with me, have this belief. This ought not to be the case. An effort to reform such a system is our obligation; it is a duty we owe to the community, to the people of the State and to the profession.

Other States are fast eliminating such objectionable obstructions to their progress, and unless we follow them we will have to contend with many undesirable influences, to which our present defective methods subject us.

Gentlemen, when we think of the rapid advancement in medical knowledge and the many changes that are so fast taking place along medical lines in other States and other countries, and what we need here in North Carolina, we naturally think of what the attitude of this society would be to such needs of reform if its policies were now coined by such men as Pittman, O'Hagan, Wood, and Thomas.

If we want to lead in medical legislation as we once did, if we wish to keep in line with medical thought, to aid in making medicine a more exact science, or if we are even content to keep in touch with other States and other countries, with the different professions and the different organizations, there must be no defect in any part of our elementary training, or of our technical growth, or of our knowledge of the basic principles of medicine or of surgery.

